# Supplementary material for: Time-Series analysis of short-term exposure to air pollutants and daily hospital admissions for stroke in Tabriz, Iran
Source: PLoS One. 2024 Nov 20;19(11):e0309414. doi: 10.1371/journal.pone.0309414 (PMC11578479; doi:10.1371/journal.pone.0309414)
Supplement: Attachment — Submitted filename: response to reviewers.docx [file pone.0309414.s002.docx]

**Response to Reviewers**:

**Title:** Time-Series analysis of short-term exposure to air pollutants and daily hospital admissions for stroke in Tabriz, Iran

Dear Prof. Phairuang,

Thank you for your consideration of the manuscript " ***Time-Series analysis of short-term exposure to air pollutants and daily hospital admissions for stroke in Tabriz, Iran*** " by *PLOS One*.

Our responses to the reviewers' queries are discussed below. A revision-marked draft of the revised manuscript is respectfully enclosed according to the instructions. In case any other revision might be required, please kindly let us know. We will do our best to make them as soon as possible.

Thank you for your consideration of the manuscript,

Sincerely,

Mahdi Banan Khojasteh PhD

**Reviewer #1:** - Your manuscript needs some revision.

Thank you for your comments and suggestions.

- The title of this study should be modified.

We changed the title in the revised version to" *Time-Series analysis of short-term exposure to air pollutants and daily hospital admissions for stroke in Tabriz, Iran* "

- Abstract should clearly inform the important findings in the present study.

- The lengthy sentences may be split in to smaller sentence without change of its meaning.

We improved this in the revised manuscript.

- Your key words seem to be general and should be revised based on MESH.

All of our current keywords are based on MESH. Per your recommendation we added more keywords.

- Background: The introduction section should be revised.

We performed a language and technical edit on the introduction for the revised version. Could you please further guide us on the specific issues in the introduction section?

- Should add some new references published in PLOS ONE.

You can add the following references:

- Borsi SH, Goudarzi G, Sarizadeh G, Dastoorpoor M, Geravandi S, Shahriyari HA, Mohammadi ZA, Mohammadi MJ. Health Endpoint of Exposure to Criteria Air Pollutants in Ambient Air of on a Populated in Ahvaz City, Iran. Frontiers in Public Health. 2022;10.

- Dastoorpoor M, Sekhavatpour Z, Masoumi K, Mohammadi MJ, Aghababaeian H, Khanjani N, Hashemzadeh B, Vahedian M. Air pollution and hospital admissions for cardiovascular diseases in Ahvaz, Iran. Science of the total environment. 2019 Feb 20;652:1318-30.

- Geravandi S, Sicard P, Khaniabadi YO, De Marco A, Ghomeishi A, Goudarzi G, Mahboubi M, Yari AR, Dobaradaran S, Hassani G, Mohammadi MJ. A comparative study of hospital admissions for respiratory diseases during normal and dusty days in Iran. Environmental science and pollution research. 2017 Aug;24:18152-9.

- Effatpanah M, Effatpanah H, Jalali S, Parseh I, Goudarzi G, Barzegar G, Geravandi S, Darabi F, Ghasemian N, Mohammadi MJ. Hospital admission of exposure to air pollution in Ahvaz megacity during 2010–2013. Clinical epidemiology and global health. 2020 Jun 1;8(2):550-6.

- Shahriyari HA, Nikmanesh Y, Jalali S, Tahery N, Zhiani Fard A, Hatamzadeh N, Zarea K, Cheraghi M, Mohammadi MJ. Air pollution and human health risks: mechanisms and clinical manifestations of cardiovascular and respiratory diseases. Toxin Reviews. 2022 Apr 3;41(2):606-17.

- Seihei N, Farhadi M, Takdastan A, Asban P, Kiani F, Mohammadi MJ. Short-term and long-term effects of exposure to PM10. Clinical Epidemiology and Global Health. 2024 May 1;27:101611.

- Abbasi-Kangevari M, Malekpour MR, Masinaei M, Moghaddam SS, Ghamari SH, Abbasi-Kangevari Z, Rezaei N, Rezaei N, Mokdad AH, Naghavi M, Larijani B. Effect of air pollution on disease burden, mortality, and life expectancy in North Africa and the Middle East: a systematic analysis for the Global Burden of Disease Study 2019. The Lancet Planetary Health. 2023 May 1;7(5):e358-69.

- Nikmanesh Y, Mohammadi MJ, Yousefi H, Mansourimoghadam S, Taherian M. The effect of long-term exposure to toxic air pollutants on the increased risk of malignant brain tumors. Reviews on Environmental Health. 2022 Jun 28.

- Hormati M, Mohammadi MJ, Iswanto AH, Mansourimoghadam S, Taifi A, Maleki H, Mustafa YF, Dehaghi BF, Afra A, Taherian M, Kiani F. Consequences and health effects of toxic air pollutants emission by industries. Journal of Air Pollution and Health. 2022 Mar 29;7(1):95-108.

- Yari AR, Goudarzi G, Geravandi S, Dobaradaran S, Yousefi F, Idani E, Jamshidi F, Shirali S, Khishdost M, Mohammadi MJ. Study of ground-level ozone and its health risk assessment in residents in Ahvaz City, Iran during 2013. Toxin reviews. 2016 Oct 1;35(3-4):201-6.

Thank you for suggesting the useful references. We read through them and used most of them to improve various sections of our paper.

- Materials and Methods: The name of study should be brought in methods section.

This is edited in the revised manuscript.

- Materials and Methods: please add the time duration of study.

The total duration of the study was 1821 days which was added to the methods section.

- Materials and Methods: Please describe how the location of sampling was selected, in details.

Location was selected based on authors presence and accessibility and availability of air pollution data. Sampling was done for all patients in Imam Rezai Hospital, Iran-Tabriz, and sampling for air pollutant variables was done using eight pollutant measurement stations in the city of Tabriz, which is mentioned in detail in the method section.

- Materials and Methods: Statistical analysis of sample data should be modified. This section is unclear.

We have added a more detailed explanation on the subject to the methods section.

- Result: The results section should be modified.

We performed a language and technical edit on this section for the revised version. Could you please further guide us on the specific issues in the introduction section?

- Result: Carefully check all Tables.

The tables were checked as instructed.

- Result: Please define the abbreviation.

This has been corrected in the revision.

- Discussion: The discussion part should be modified.

We revised and expanded on some parts of the discussion in our revised manuscript. If there is anything else we can do to improve our work please let us know.

- Discussion: Refer to more updated articles on similar studies in the discussion section and also the reference list.

To write the discussion section, similar studies were selected by careful search, and among the reviewed articles, the most recent studies that were comparable to our results were selected and discussed. The studies reviewed in the discussion section were the most recent studies that we could discuss and compare with our study.

- Discussion: Please highlight your study's strengths and limitations.

We added more explanation to the relevant sections in the revised manuscript.

- Discussion: Suggest adding a paragraph on directions for future research, practice, and policy.

We added suggestions for future research based on our limitation to the discussion. We also added practice and policy suggestions as requested.

- Conclusions should be short with important observations.

We improved our conclusion for the revision.

**Reviewer #2:** Comment to authors

I would like to congratulate on the effort in writing the article: "Air Pollution and Daily Hospital Admissions of Stroke Patients: A time-series analysis of exposure in Tabriz, Iran".

I must say that this article is very interesting and will contribute to know the usefulness of time series studies.

I left you some observations to improve the quality of the information you try to communicate:

Thank you for your precise and constructive suggestion. We greatly appreciate your attention and time to our manuscript.

Title: I think you need to include in the title: Short-term associations

Short-term associations of air pollution and daily hospital admissions in stroke patients: A time-series analysis of exposure in Tabriz, Iran

We revised the title to be more fluent and include “short-term” phrase in it.

Introduction:

The authors say:

“The most recent umbrella review and meta-analysis studies in this field indicate a significant association between six major air pollutants and stroke incidence, stroke hospitalization, and stroke mortality [16, 25].”

Please specify which six major air pollutants.

The pollutants were PM2.5, PM10, NO_2_, SO_2_, O_3_, CO which is added in the revision.

Methods

1.- Please state the number of cases included.

The total number of cases were 4865 which is added into the revised manuscript.

2.- When you use ICD 10 you say:

The inclusion criteria for this category were defined by diagnostic ICD 10 codes falling within 161 to 164.

Please check, because I think the code is I61 to I64. With (I) before nor number 1 before.

We corrected the mistake in the revision.

Please specify why you don't analyse different types of stroke.

The included ICD-10 codes cover both ischemic and hemorrhagic strokes. Analysis of different types of stroke was not amongst our goals and as such we don’t have data on subtypes.

Exposure data:

Please specify outcome variable: I assume that the outcome variable is hospital admissions of stroke patients, but you don't specify this variable in the text, please clarify e.g. whether the outcome variable is admissions or hospitalised patients and why you don't use hospitalised patients.

We have two hospitals with neurology wards for stroke patients (Imam Reza and Razi). All patients suspected of neurological problems are first brought into Imam Reza Hospital. They are visited by a neurologist and given a diagnosis. if the patient is still in the golden window for tTPA treatment, they remain hospitalized in Imam Reza to receive treatment. The remaining patients are sent to Razi Hospital. Hence, our stroke patients all receive admission to Imam Reza Hospital but are not necessarily hospitalized there due to the limited capacity of the wards. More explanation was added to the methods section.

In the methods, you must specify the reason why you are using the ARIMA model and not another model, or classical analysis of time series studies and the reason why you are using the ARIMA model with this description as (3,1,3) each parameter has a reason, please specify.

A cross correlation graph may be useful.

More details on the statistical methods have been added to the revised manuscript.

Limitations:

It is necessary to include in the limitations the possibility that your study is affected for Berkson bias because it is not a community study, please review about this bias and include this information in the limitations part.

Please include in the limitations that your study can't be extrapolated to other realities.

You may recommend that this study should at least be done in the community to contrast the results.

We have revised the limitation section as suggested to be more comprehensive and include the aforementioned bias as well.

**Reviewer #3:** This study lack of novelty. novelty of the study should be highlighted.

Thank you for your precise comments and consideration.

The primary novelty of our study is the location of our study. This is especially important since very few studies on the subject come from low-middle income countries and the Middle East which suffer a great burden of stroke. Furthermore, many studies on the subject did not use time series analysis in their statistical methods.

In our revised manuscript, we have added a section to the introduction and discussion.

Title needs to be rewritten.

We changed the title in the revised version to" *Time-Series analysis of short-term exposure to air pollutants and daily hospital admissions for stroke in Tabriz, Iran* "

The implemented model id ARIMA or ARIMA-X model.

The ARIMA-X model was utilized in this study.

Necessary equation and flowchart of the model should be included.

The requested changes have been added to the revised manuscript.

Detailed methodology should be provided.

More detail on the subject has been added to the relevant section.
